# Hypothermia in a Rural Setting: An Emergency Medicine Simulation Scenario

**DOI:** 10.7759/cureus.1998

**Published:** 2017-12-28

**Authors:** Felix Zhou, Robert Jong, Aron Heroux, Adam Dubrowski

**Affiliations:** 1 Medical Education, Memorial University of Newfoundland; 2 Medicine, Memorial University of Newfoundland/Janeway; 3 Locum Physician; 4 Emergency Medicine, Pediatrics, Memorial University of Newfoundland

**Keywords:** hypothermia, rural, simulation, emergency medicine

## Abstract

Patients presenting with hypothermia in a rural emergency department can be quite challenging to manage without significant mortality and morbidity. Standard medical school curricula do not fully prepare trainees for the unique aspects of practice in northern rural and remote communities. Training opportunities on site may provide a solution to this lack of experience. However, these communities often have limited simulation-based resources and expertise for conducting and developing simulation scenarios. In this technical report, we outline a hypothermia simulation that utilizes only basic resources and is, thus, practical for rural and remote facilities. The aim of this report is to better equip trainees, clinicians, and emergency department staff who may encounter such a scenario in their practice. While the simulation is specifically designed for medical students, resident doctors, and emergency department staff, it could also be applicable in other low-resource settings, such as military bases, search and rescue stations, and arctic travel and tourism infirmaries.

## Introduction

Exposure to harsh winter conditions can present a significant risk to the inhabitants of northern rural and remote communities. Accidental hypothermia is associated with significant morbidity and mortality [1]. While there is relatively little published data on the prevalence of hypothermia across Canada, a study conducted in British Columbia described more than 350 deaths with the underlying or contributing cause being hypothermia during a 14-year period [2]. Interestingly, in a single tertiary center, there were 14 different rewarming strategies used for roughly 80 cases of hypothermia [3]. This suggests a lack of consistency in the management of hypothermia and could lead to uncertainty and delayed decision-making for medical professional trainees. Because of the relatively rare occurrence and high morbidity and mortality of hypothermia, emergency department staff need to be able to appropriately treat it in a timely manner. Simulation can help fill the gaps in the undergraduate curriculum for northern rural and remote practice as well as provide a refresher for trained physicians who may be new to rural and remote care in the North.

This technical report re-creates the arrival of a patient in a moderately hypothermic state (defined as a core body temperature of 28°C to 32°C). The objective of this simulated training exercise is to provide learners with the following knowledge and skills:

1)  Addressing the circulation, airway, breathing, disability, exposure (CABDE) priorities

2)  Appropriately measuring the core temperature

3)  Administering appropriate rewarming techniques

4)  Recognizing afterdrop

5)  Managing arrhythmias and other complications of hypothermia

This simulation session is appropriate for the training of third- or fourth-year medical students, residents, and staff physicians. Other medical professionals are encouraged to participate in the simulation for team building and practice.

## Technical report

All elements, such as the educational context, inputs, processes, and expected products related to the development and implementation of this simulation case, are organized following a modified context, input, process, product (CIPP) model program development and evaluation model [4].

Context

Simulation training may take many forms, from imaginative scenario-based work (usually cases discussed on paper) to individual or team-based manikin or standardized patient scenarios, to the acquisition of technical skill on task-based trainers. These types of simulations can be delivered in a hospital or medical school-based simulation center or in a real clinical setting (which is known as an in-situ simulation).

The simulation described in this technical report can be run in an emergency department (ED) trauma bay, medical school or hospital-based simulation center, or a combination of these settings. The staff or resident doctor should be the main learner, with medical students, nurses, other physicians, paramedics, or respiratory therapists assisting as needed.

Inputs

The following list of equipment is required to run the simulation:

1.  Low fidelity manikin; we recommend the Resusci Anne First Aid manikin ( Laerdal Medical, Stavanger, Norway) [5] with a vital sign monitor.

2.  Two emergency department (ED) nurses, if available. If ED nurses are unavailable, other learners on the team can act these roles.

3.  Simulation facilitator(s), who will provide supporting information (ex. vitals, electrocardiograms (ECGs)), ensure adherence to the template, and assess individual and team performance.

4.  A printed storyboard and objective summary sheet for the facilitator to follow along and provide the learner with vital information as the case progresses.

5.  Four liters (L) of normal saline.

6.  Two large (14 or 16 gauge) peripheral intravenous (IV) catheters and a Foley catheter.

7.  Microwave.

8.  Monitor/defibrillator.

9.  An esophageal probe or low-reading thermometer.

10.  Bair Hugger Company, (3M Company, Maplewood, Minnesota, United States) [6] or blankets.

Process

The facilitator should print this report and separate the storyboard and table summarizing the objective highlights. These will act as the template to help guide the simulation session as it progresses. The facilitator can also read the in-depth objective descriptions within the report for an explanation of the interventions.

Before the case starts, the facilitator will designate the roles of simulation leader and supporters. The facilitator will address issues of confidentiality and comfort and seek an agreement to the fiction contract with all who are involved. The scenario begins with the leader having the case read to them and learning that they have a hypothermic patient about to arrive. The leader will be asked by the facilitator to verbalize what medical issues they anticipate, and when ready, the team will begin the simulation. As the simulation progresses, the facilitator should follow along on the storyboard and objective summary sheets, updating the simulation leader as they progress. Once the case progresses to the end of the storyboard (as the patient stabilizes), the participants should begin the formal debriefing session.

Products

The expected products are organized according to the CanMEDS roles [7].

Medical Expert: The management of hypothermia involves clinical decision-making, interpreting diagnostic tests, procedural skill proficiency, and adapting to new findings as they present.

Collaborator and Communicator: This scenario promotes the use of closed-loop communication while working within the healthcare team with a clear definition of roles. If there is any question on how to proceed, the trainee should elicit advice from team members and allied health staff.

Case

A 32-year-old male is brought in by emergency medical services (EMS) after being retrieved from a snowbank at 8 am. He has significantly altered mental status but is not shivering. The patient had been snowmobiling late the night before and was found the next morning by his wife who called for help. An unknown amount of alcohol had been consumed. The weather last night was -5 °C with 15 km/h winds. The patient was taken by EMS to the local emergency department. The emergency medical team and an ED nurse are immediately available with a lab and radiology technician available upon request.

The patient's wife provided a collateral history that revealed no allergies, no medications, a pack-a-day smoker for 15 years, and no pertinent past medical history. The patient’s skin and mucous membranes showed prominent central and peripheral cyanosis. There are no obvious signs of trauma; however, the patient is in a c-spine collar. The patient’s last meal was around 12 hours ago, and he was expected to have been in the snowbank for somewhere between six and eight hours.

The initial vitals were recorded as follows:

·  Heart rate: 40

·  Blood pressure: 70/40 mmHg

·  Oxygen saturation (SpO2): 80%

·  Respiratory rate (RR): 6

Following this information, the leader should initiate and complete the following objectives.

Objectives

**Figure 1:**
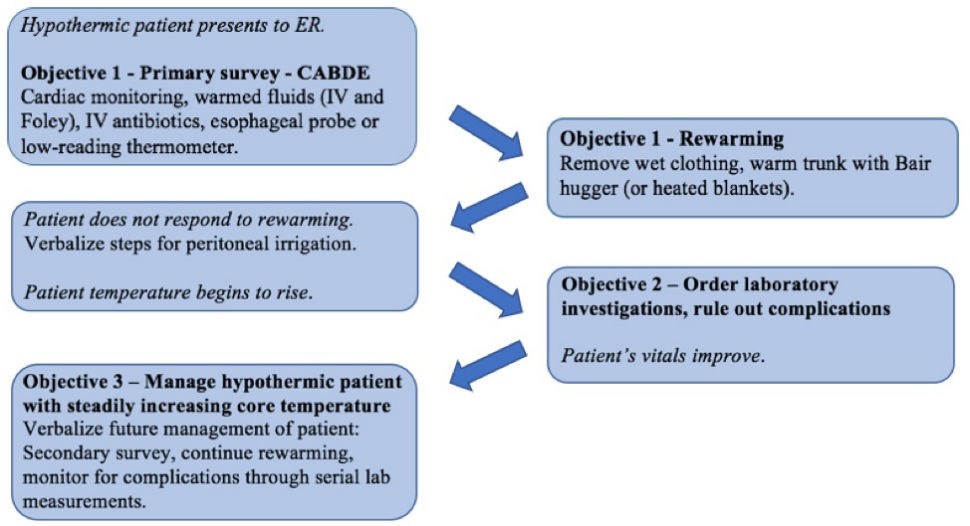
Storyboard representation of the course of the simulation as the leader progresses through each objective.

See Figure 1 for an overview of the simulation presented as a storyboard, and Table 1 for a summary of the objectives/expected actions/vitals/cues, which the facilitator may use as a reference during the simulation.

**Table 1:** Summary of the expected actions (organized by learning objectives) that the leader must progress through during the simulation, along with the appropriate vital signs at each step. To the right of the table are various cues with which the facilitator may prompt the simulation leader to steer the leader back to the appropriate expected actions.

Objective 1: Recognize a situation of severe hypothermia. *Perform primary survey *(tailored for hypothermia) and begin rewarming.		
Vital signs	Expected actions	Cues
Heart rate (HR): 40. Blood pressure: (BP): 70/40. Oxygen saturation (SpO_2_): 80%. Respiratory rate (RR): 6. Core body temperature: 30°C.	Primary survey. Circulation: Recognize the potential for arrhythmias/cardiac arrest, and attach to monitor/defibrillator. Recognize hypotension. Give isotonic crystalloid (warmed to 42°C) through two, large, peripheral IV catheters and a Foley catheter at a rate of 1 L/hour (hr). Administer intravenous (IV) antibiotics (1 gram ceftriaxone). Airway/Breathing: Look, listen, feel. Bradypneic (RR = 6), decreased breath sounds and chest movements. The leader should not intubate. Insert an esophageal probe or a low-reading thermometer. Disability: Address Glasgow Coma Scale (GCS). Exposure: Assess for fractures/lacerations.	Ask the learner to address the primary survey. Circulation: Once the monitor/defibrillator is attached, may provide the leader with an electrocardiogram (ECG) showing atrial fibrillation. Airway/Breathing: If the leader begins to intubate, ask the leader to verbalize the benefits and risks of intubating in this scenario. When the esophageal probe/low-reading thermometer has been inserted, state that body temperature = 30°C. Disability: State the patient’s GCS score aloud (GCS = 6). Exposure: State that the patient has no fractures/lacerations.
Objective 1: Recognize the situation of severe hypothermia. Perform the primary survey (tailored for hypothermia) and begin rewarming.		
Vital signs	Expected actions	Cues
Same as above	Rewarming should occur simultaneously with the primary survey. Passive external rewarming technique: Remove all wet clothing. Active external rewarming technique: Bair Hugger [6] or heated blankets. Warm trunk before limbs to avoid afterdrop. Active internal rewarming techniques: Heated fluids (intravenous (IV) and Foley); less invasive, done during the primary survey. Once prompted by the facilitator, the leader will verbally explain peritoneal irrigation.	Following the administration of passive external, active external, and heated fluids, the leader may wait to see if the patient’s core temperature rises. The facilitator should state that the patient fails to respond, and ask the leader to verbalize the next rewarming technique (peritoneal irrigation). Upon explanation, the facilitator will state that the patient’s temperature is increasing.
Objective 2: Order appropriate laboratory investigations for the further management of the hypothermic patient. Rule out potential complications of hypothermia/rewarming.		
Vital signs	Expected actions	Cues
HR: 50. BP: 90/60. SpO_2_: 90%. RR: 12. Temperature: 32°C.	Order the following labs: Fingerstick glucose, arterial blood gas, complete blood count (CBC), electrolytes, blood urea nitrogen (BUN), creatinine, coagulation studies, serum toxicology screen (benzodiazepines, cocaine, etc.), and blood alcohol content. Once the leader is asked by the facilitator to explain the significance of these results, the leader should explain that hypoglycemia, acid-base abnormalities, infection, electrolyte abnormalities, and kidney damage are ruled out.	Once labs are ordered, verbalize the results: All tests are normal, except the blood alcohol content elevated (0.045 g/dL). Blood gas shows pH: 7.4, PCO_2_: 40 mmHg, HCO3^-:^ 24 mmol/L, and PaO_2_: 60 mmHg. Subsequently, ask the leader to state the significance of these tests.
Objective 3: Manage the hypothermic patient with a steadily increasing core temperature		
Vital signs	Expected actions	Cues
HR: 60. BP: 110/90. SpO_2_: 93%. RR: 16. Temperature: 34°C.	Verbalize the plan for future management: Secondary survey. Continue rewarming. Monitor for complications (e.g. hypotension, electrolyte abnormalities, etc.) through iterative clinical evaluation and serial lab measurements.	Once the leader has achieved the previous objectives, state the new set of vitals aloud. Subsequently, ask the leader about the future plan for the management of this patient. Once the plan has been verbalized, the facilitator may verbalize the conclusion of the simulation.

Objective 1: Recognize a situation of hypothermia. Perform the primary survey (tailored for hypothermia) and begin rewarming.

Primary Survey

**Figure 2:**
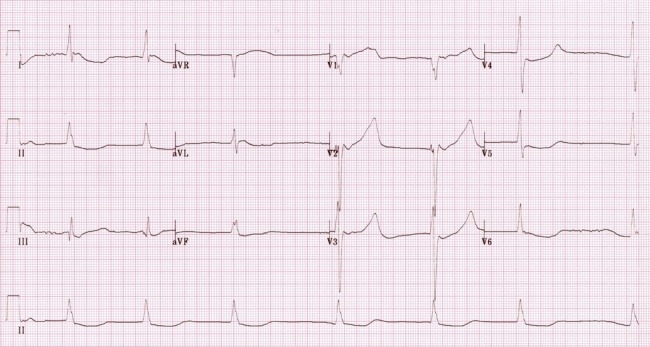
ECG showing atrial fibrillation with slow ventricular response in a moderately hypothermic patient. Taken from https://lifeinthefastlane.com/wp-content/uploads/2011/03/slow-AF-hypothermia.jpg. ECG: electrocardiogram

In the management of hypothermia, the primary survey is slightly modified from ABCDE to CABDE. Beginning with circulation, the simulation leader should recognize that a moderately hypothermic patient with a weak pulse should undergo continuous cardiac monitoring and may descend into lethal arrhythmias (including ventricular fibrillation) or even cardiac arrest. Accordingly, the patient should be attached to a combined monitor/defibrillator. The monitor will show that the patient is in atrial fibrillation. If prompted, the simulation facilitator may provide the patient with an electrocardiogram (ECG) of the moderately hypothermic patient (Figure 2).

The leader should recognize that atrial fibrillation is a common arrhythmia in hypothermia and often resolves spontaneously with rewarming; no specific intervention is needed [1]. Finally, the leader should recognize the potential for severe hypotension once rewarming commences and give warmed infusions of isotonic crystalloid through two, large (14 or 16 gauge), peripheral IV catheters (4 L) at a rate of 1 L/hr. In addition, a Foley catheter should be inserted. Like a hospital in a rural community, the bags of fluid will be warmed to 42°C in a microwave, which will have set times posted on the door (in this simulation, we will use 90 s). Intravenous (IV) antibiotics (1 gram ceftriaxone) will be given once as well, as infection cannot be ruled out at this point.

Next, the simulation leader should evaluate the airway. The leader will look, listen, and feel, recognizing that the patient is bradypneic (respiratory rate (RR) = 6) with decreased breath sounds and chest movements. The leader should defer intubation and continue on with the rest of the primary survey. If the leader begins to intubate, the facilitator should ask the leader to verbalize the benefits and risks of intubation in this scenario of a hypothermic patient with bradypnea and decreased level of consciousness. Intubation secures the airway, and in patients with cold-induced bronchorrhea, intubation may help facilitate the clearance of airway secretions. However, intubation may delay rewarming, and the administration of a paralytic agent suppresses the natural shivering response, which becomes an important source of thermogenesis as the patient progresses from moderate to mild hypothermia (at 32°C). If the patient's level of consciousness remained depressed after initial resuscitation or if initial lab results suggested sepsis, it would then be reasonable to intubate. However, there are relatively few benefits to early intubation in this scenario, as most rural centers do not have the ability to administer warmed air through a ventilator.

The leader will measure the core temperature of the patient with an esophageal probe, inserted into the lower one-third of the esophagus (~24 cm below the larynx; proper placement is estimated using the measurement marks on the probe). A low-reading thermometer may be used instead if an esophageal probe is not available. Once inserted, the facilitator will verbally state the body temperature of the patient (30°C). The leader should address the patient’s level of consciousness using the Glasgow Coma Scale (GCS). The facilitator will state the patient’s GCS score aloud (GCS = 6). Finally, the leader will verbally ask the facilitator if the patient has any fractures, lacerations, or other injuries outside of the hypothermia. The facilitator will confirm the absence of any external injuries, thus completing the primary survey.

Rewarming

While the primary survey is underway, the simulation leader should simultaneously begin the rewarming of the patient. To begin, all wet clothing should be removed. Next, the simulation leader should employ active external rewarming techniques by placing the patient in the Bair Hugger [6], a machine that utilizes forced-air warming. If the simulation takes place in a rural center where a Bair Hugger [6] is not available, other active external rewarming techniques may be used instead (e.g. heated blankets). Whichever method is used, the leader should ensure that the patient’s trunk is warmed before their limbs, in order to minimize core temperature afterdrop, the phenomenon where core temperature drops during rewarming due to a dilation of peripheral vessels and the subsequent return of cool blood to the core. Afterdrop may lead to severe hypotension and lethal arrhythmias.

Following the application of passive external rewarming techniques (removing wet clothing), active external rewarming techniques (Bair Hugger [6] or warmed blankets), and less invasive, active, internal rewarming techniques (heated IV fluids), the leader may wait to see if the patient’s core temperature rises accordingly. If this occurs, the facilitator should state that the patient fails to respond to these interventions, and ask the leader to verbalize the next rewarming technique that may be applied. In response, the leader should describe the steps involved in peritoneal irrigation. In peritoneal irrigation, two catheters (one for instillation and one for drainage) are placed in the infraumbilical region. Subsequently, 10 to 20 ml/kg of warmed isotonic saline are infused and left in the peritoneal cavity for 20 minutes, and then removed. The overall exchange rate is 6 L/hour [8]. The facilitator will state that the patient’s core temperature is rising appropriately in response to current rewarming techniques.

Objective 2: Order appropriate laboratory investigations for the further management of the hypothermic patient. Rule out the potential complications of hypothermia/rewarming.

Following the primary survey and initiation of rewarming techniques, the leader should order a finger-stick glucose, arterial blood gas, complete blood count (CBC), electrolytes, blood urea nitrogen (BUN), creatinine, coagulation studies, serum toxicology screen (benzodiazepines, cocaine, etc.), and blood alcohol content. The facilitator will verbalize the results: all tests are normal, except the blood alcohol content is elevated (0.045 g/dL). Arterial blood gas shows pH: 7.4, PCO_2_: 40 mmHg, HCO3^-^: 24 mmol/L, and PaO_2_: 60 mmHg. The facilitator will prompt the leader to verbalize the significance of these results with regards to potential complications and the impedances of rewarming, namely that hypoglycemia, acid-base abnormalities, infection, electrolyte abnormalities, and kidney damage are ruled out.

Objective 3: Manage the hypothermic patient with a steadily increasing core temperature.

Once the previous objectives are achieved, the facilitator will state a new set of vitals. To conclude the simulation, the facilitator will ask the leader about their plan for the future management of this patient. The leader should verbalize their intent to perform a secondary survey, continue rewarming, and monitor for complications (e.g. hypotension, electrolyte abnormalities, rhabdomyolysis, disseminated intravascular coagulopathy, acute renal failure, and pneumonia/septicemia) through an iterative clinical evaluation and serial lab measurements.

Debriefing

After the scenario, a formal debriefing is conducted with the learners. To establish an environment of psychological safety conducive to learning, the simulation facilitator should clarify the confidential nature of the debriefing and reaffirm their belief in the learner’s ability, commitment to doing their best, and desire to improve [9-10]. A summary is listed here:

            1) Observe the gap between desired and actual performance.

            2) Provide feedback about the performance gap.

            3) Investigate the basis for the performance gap.

            4) Help close the gap through discussion and didactics.

Post-scenario didactics

After the debriefing session, it is advised to conduct a brief didactic session in which the educators can address any knowledge gaps identified during the scenario and subsequent debrief. This allows the trainees to consolidate new knowledge obtained during the simulation. In addition, the facilitator may discuss the possibility of prolonged resuscitation (exceeding several hours) in cases of hypothermia, as well as the numerous biochemical markers that are indicators of a poor prognosis, including hyperkalemia, hypernatremia, and elevated ammonia and lactate [11-12].

## Discussion

The aim of this simulation is to educate learners on an approach to managing a hypothermic patient in a rural hospital setting by providing an appropriately challenging learning experience. Specifically, learners will utilize a stepwise approach to the primary survey and rewarming, order appropriate laboratory investigations, and rule out the complications of rewarming. This stepwise approach has been modeled after two review articles from the New England Journal of Medicine [1,8], with slight modifications to the protocol based on some of the unique aspects of rural medicine. For example, rural facilities may not have the ability to provide heated air via ventilators, and they may not have Bair Hugger machines readily available for active external rewarming. This report offers reasonable, practical solutions to these barriers.

Patients presenting with hypothermia are a regular occurrence in northern rural health care facilities. Therefore, appropriate training for the management of these patients is essential for rural physicians and trainees. Furthermore, given that rural health care facilities may have reduced resources for the management of these patients, this scenario is also useful for teaching physicians to work with limited resources and smaller teams. In these situations, where evidence-based protocols cannot be followed exactly as written, clinical decision-making becomes important. This report provides a rational approach to the management of hypothermia in settings where specific therapies are not available. In addition, this simulation requires relatively inexpensive and few resources, thus, it may be easily incorporated into the curricula of medical schools. Regardless of the learner’s level of training, after completing this scenario, the learner should have a better understanding of the approach to managing hypothermic patients as well as some complications that may impede this.

In addition to the simulation portion of this exercise, learners will also have an opportunity to receive feedback on their performance, as well as reflect on their management of the simulated patient. This debriefing session is essential for reinforcing the correct technique, as well as identifying the areas in which they should improve.

## Conclusions

Rural and remote medical practice can be difficult in some situations, such as the management of hypothermia. The standard undergraduate curriculum may not prepare students for these unique challenges. Based on the lifestyle (e.g. outdoor activities), geography, support, and climate in northern remote locations, physicians will likely manage a hypothermic patient at some point in their career. These can be complicated and intimidating cases. After completing this simulation, learners should have an improved understanding and confidence in the management of these situations with effective communication and collaboration. Participating in this case will provide a memorable learning experience that can refresh and help solidify clinical management strategies. While this case is designed to be run in a rural emergency room, it could also be quickly modified to suit any low-resource environment, such as military, search and rescue, or arctic travel and tourism.
